# Profile of Circulatory Cytokines and Chemokines in Human Coronaviruses: A Systematic Review and Meta-Analysis

**DOI:** 10.3389/fimmu.2021.666223

**Published:** 2021-05-05

**Authors:** Ayat Zawawi, Abdallah Y. Naser, Hassan Alwafi, Faisal Minshawi

**Affiliations:** ^1^ Department of Medical Laboratory Technology, Faculty of Applied Medical Sciences, King Abdulaziz University, Jeddah, Saudi Arabia; ^2^ Department of Applied Pharmaceutical Sciences and Clinical Pharmacy, Faculty of Pharmacy, Isra University, Amman, Jordan; ^3^ Faculty of Medicine, Umm Al-Qura University, Makkah, Saudi Arabia; ^4^ Department of Laboratory Medicine, Faculty of Applied Medical Sciences, Umm Al-Qura University, Makkah, Saudi Arabia; ^5^ Vaccines and Immunotherapy Unit, King Fahad Medical Research Center, King Abdulaziz University, Jeddah, Saudi Arabia

**Keywords:** cytokine storm, inflammatory cytokines, COVID-19, MERS, SARS, SARS-CoV-2

## Abstract

**Background:**

SARS, MERS, and COVID-19 share similar characteristics. For instance, the genetic homology of SARS-CoV-2 compared to SARS-CoV and MERS-CoV is 80% and 50%, respectively, which may cause similar clinical features. Moreover, uncontrolled release of proinflammatory mediators (also called a cytokine storm) by activated immune cells in SARS, MERS, and COVID-19 patients leads to severe phenotype development.

**Aim:**

This systematic review and meta-analysis aimed to evaluate the inflammatory cytokine profile associated with three strains of severe human coronavirus diseases (MERS-CoV, SARS-CoV, and SARS-CoV-2).

**Method:**

The PubMed, Embase, and Cochrane Library databases were searched for studies published until July 2020. Randomized and observational studies reporting the inflammatory cytokines associated with severe and non-severe human coronavirus diseases, including MERS-CoV, SARS-CoV, and SARS-CoV-2, were included. Two reviewers independently screened articles, extracted data, and assessed the quality of the included studies. Meta-analysis was performed using a random-effects model with a 95% confidence interval to estimate the pooled mean of inflammatory biomarkers.

**Results:**

A high level of circulating IL-6 could be associated with the severity of infection of the three coronavirus strains. TNF, IL-10, and IL-8 are associated with the severity of COVID-19. Increased circulating levels of CXCL10/IP10 and CCL2/MCP-1 might also be related to the severity of MERS.

**Conclusion:**

This study suggests that the immune response and immunopathology in the three severe human coronavirus strains are somewhat similar. The findings highlight that nearly all studies reporting severe cases of SARS, MERS, and COVID-19 have been associated with elevated levels of IL-6. This could be used as a potential therapeutic target to improve patients’ outcomes in severe cases.

**Systematic Review Registration:**

PROSPERO registration 94 number: CRD42020209931.

## Introduction

Coronavirus disease 2019 (COVID-19) is the third human coronavirus (hCoV) outbreak in only two decades (1). Coronaviruses (CoVs) are enveloped, single-stranded RNA viruses that infect the lower respiratory tract (2). Severe acute respiratory syndrome (SARS), caused by SARS-CoV, was the first global epidemic of the twenty-first century (3, 4). In March 2003, the disease broke out in Hong Kong, quickly spreading throughout the world, including Asia, Europe, and the United States (5). Nearly a decade following the SARS epidemic, in June 2012, Middle East respiratory syndrome (MERS), caused by MERS-CoV, emerged in Saudi Arabia and rapidly spread to more than 20 countries (6). Bats are known to be the natural reservoir of SARS and MERS. Dromedary camels have also been found to be a zoonotic reservoir for transmission of MERS to humans (7).

COVID-19 is the newest emerging infectious disease (8). The infection began in Wuhan, China, in December 2019 and has infected over 100 million people, with over 2 million deaths worldwide as of April, 2021 (9). This version of human coronavirus is a more serious global threat than either of its two predecessors.

The three human coronaviruses share similar characteristics; the genetic homology of SARS-CoV-2 to SARS-CoV and MERS-CoV is 80% and 50%, respectively (10). All three respiratory infectious diseases are also associated with significant morbidity and mortality, as they are highly transmissible, primarily through respiratory droplets and close contact (11). However, the transmission of MERS to humans is relatively inefficient compared to SARS and COVID-19 (12, 13). Conversely, SARS and COVID-19 have a relatively lower mortality rates than MERS (14). This is likely related to the viral kinetics, age and comorbid illnesses (14).

The three respiratory infectious diseases (SARS, MERS, and COVID-19) have similar clinical features that range from a lack of symptoms to severe illness that requires intubation and intensive care management (15, 16). The severity of these diseases is linked to age and chronic illness, including diabetes, hypertension, cardiovascular disease, chronic respiratory disease, and cancer (17). Viral pneumonia, acute respiratory distress syndrome, and multiple organ failure are common in these diseases’ later stages (18–20). Non-specific laboratory tests, including those for leukopenia, lymphopenia, thrombocytopenia, and elevated serum amino transaminases, are often positive in SARS, MERS, and COVID-19 infections (21).

**Figure 1:**
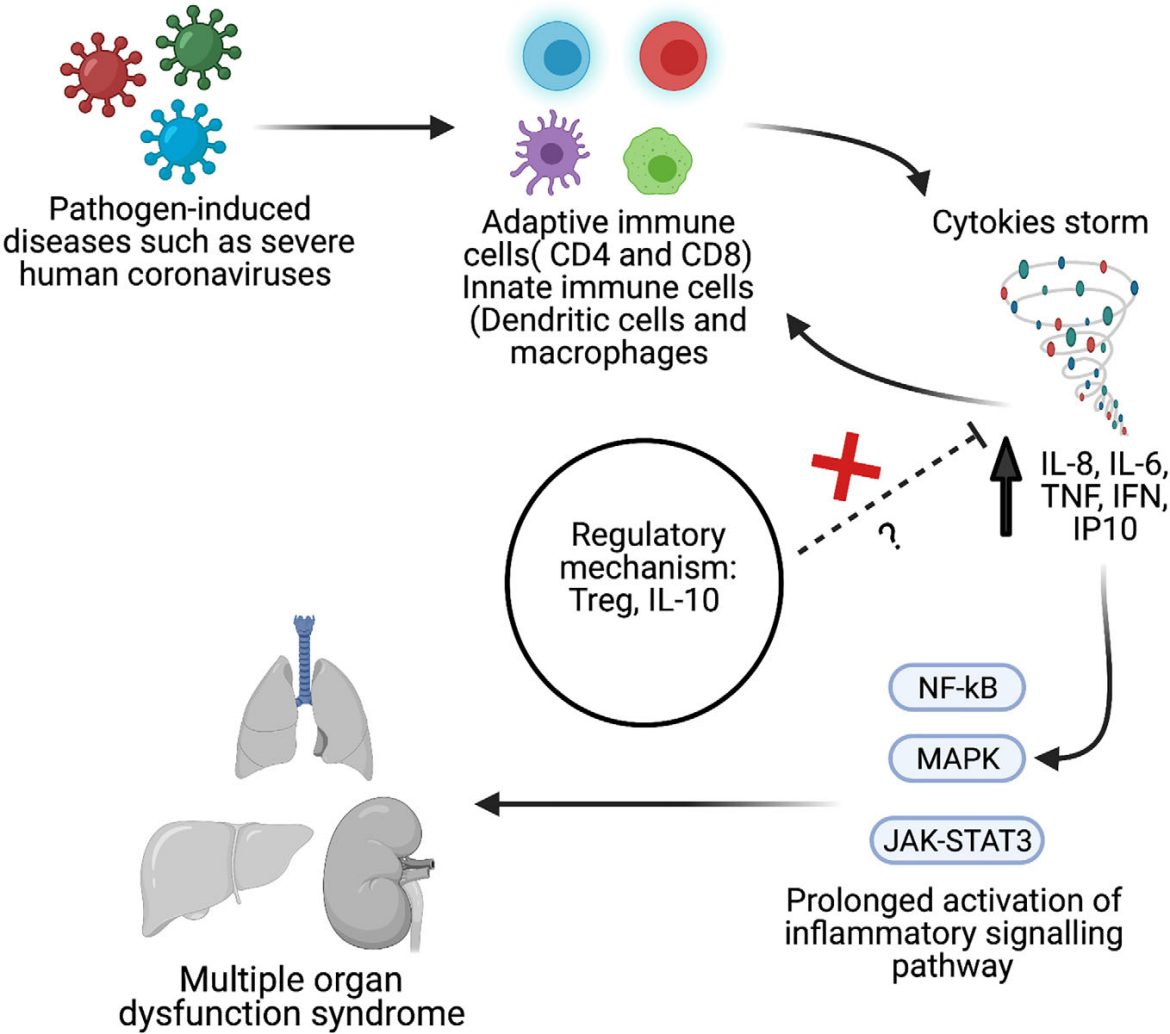
Schematic overview of pathological hallmark of cytokines storm during viral infection such as severe human coronavirus (hCoV). Created with BioRender.com.

A cytokine release storm (CRS) is the uncontrolled release of several proinflammatory cytokines due to an exuberant host immune response (22–24). In SARS (25), MERS (26), and COVID-19 (27, 28) cases, CRS has been reported to lead to the development of severe phenotypes (**Figure 1**). Given the potential overlap in presentation and manifestation among severe hCoV infections, and the absence of effective treatment, it is essential to understand hCoVs’ immunopathology, including hCoV-induced CRS. Therefore, this systematic review and meta-analysis aimed to compare the inflammatory biomarkers associated with three significant strains of severe human coronavirus disease (MERS-CoV, SARS-CoV, and SARS-CoV-2) that may have a distinct inflammatory profile. Such results could help to identify potential treatment options for severe patients.

## Methods

The systematic review and meta-analysis were carried out following the Meta-analysis of Observational Studies in Epidemiology (MOOSE) guidelines (29) and reported following the Preferred Reporting Items for Systematic Reviews and Meta-Analyses (PRISMA) statement (30). The protocol of the study was registered with PROSPERO (provisional registration number CRD42020209931).

### Databases and Search Strategy

**Figure 2:**
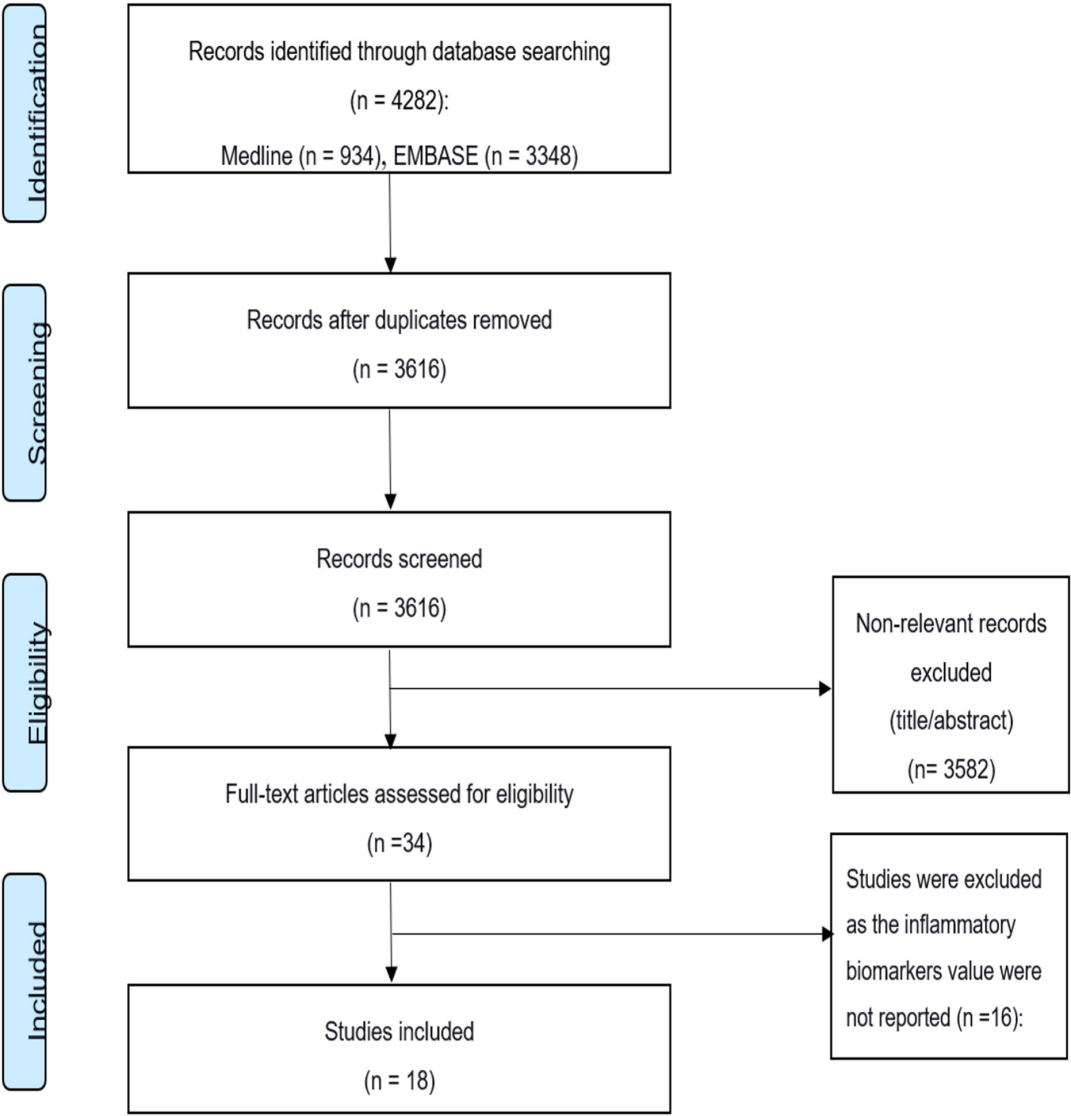
The PRISMA flow diagram of study inclusion/exclusion process.

An extensive search strategy (summarized in **Figure 2**) was developed to identify relevant studies. A detailed electronic search on bibliographic databases, including Medline, Embase, and Cochrane Library, was performed from inception to July 2020. Keywords, Emtree, and MeSH terms were used with both English and American spellings. The search strategy covered the following keywords: cytokines, inflammatory biomarkers, SARS-CoV, SARS-CoV-2, severe acute respiratory syndrome, Middle East respiratory syndrome coronavirus, and COVID-19.

### Eligibility Criteria

The selection of included studies was based on inclusion/exclusion criteria. Inclusion criteria include any randomized or observational studies that assessed the concentration of cytokines and chemokines in the peripheral blood of patients with severe SARS, MERS, or COVID-19 infections. Severe or critical patients were defined as patients who had a respiratory failure, required mechanical ventilation, experienced shock, experienced other organ failures, or required a stay in the intensive care unit (ICU). Conference proceedings, reviews, studies not published in English, animal studies, and *in vitro* studies of cytokine production in stimulated cells were excluded. Studies that did not clearly differentiate between levels of severity or did not report the mean and standard deviation or median of cytokine parameter levels were also excluded.

### Data Extraction

Initially, all the researchers (A.Z., A.N., H.A., and F.M.) independently screened the title and abstract of each study to assess its eligibility based on the inclusion/exclusion criteria. Subsequently, all the researchers (A.Z., A.N., H.A., and F.M.) independently conducted a two-step full-text literature search to further assess eligibility. Data extraction from the relevant studies was performed independently by all researchers using a form created for this study. The following demographic details were extracted from each study: study type, data source, year of publication, sample size, age, and sex. The mean, median, standard error, IQR, and range of certain cytokines and chemokines (IL-1, IL-2, IL-4, IL-6, IL-8, IL-10, IL-17, TNF, IFN-γ, RANTS, CCL2/MCP-1, CXCL10/IP10, and TGFß) were also extracted.

### Quality Assessment

The methodological quality and risk of bias were assessed using the Newcastle Ottawa Scale for Observational Studies (31), which was modified to meet the requirements of this study (32). A total of six criteria were evaluated: representativeness of the population, sample size, statistical analysis, missing data, methodology for reporting the outcome of interest, and methods to detect or report the outcome of interest. Each criterion was rated on a scale ranging from 0 to 3, where 3 represented the highest quality. The highest possible total score was 18. In addition, we categorized the score into three categories: good quality (>12), moderate quality (>6), and low quality (≤6).

### Data Synthesis and Analysis

Descriptive statistics were used to describe the demographic characteristics and quantitative mean concentration of the inflammatory biomarkers reported in each study. To standardize the data for meta-analysoo, we estimated the mean cytokine concentrations from studies that reported the median using the following formula: if the sample size was ≤25, $\left(X=\frac{\text{a}+2\text{m}+\text{b}}{4}\right)$, and if the sample size was >25, (). We calculated the variance as follows: if the sample size was ≤15, $\left(S^{2}=\frac{1}{12}\left[\frac{\left(a-2m+b\right)}{4}{\left(b-a\right)}^{2}\right]\right)$; if the sample size was 15–70, $\left(S=\frac{R}{4}\right)$; and if the sample size was 70, $\left(S=\frac{R}{6}\right)$, where m is the median, a is a minimum, b is a maximum, X is a mean, S is variance and R is a range (b-a) (33). A random-effects model was used to estimate the mean concentration of biomarkers (34). Heterogeneity among the studies included in the meta-analysis was assessed using the standard χ2 tests and the I^2^ statistic. If high heterogeneity was indicated (I^2^ ≥ 75%), systematic narrative synthesis was provided. All analysis was performed using STATA 15.0.

## Results

### Study Selection, Characteristics, and Quality Assessment

A total of 4,282 studies were identified through the initial online search of the databases (**Figure 2**). Of these, 666 studies were duplicates. Title and abstract screening identified 34 studies for final full-text review using the inclusion/exclusion criteria. Finally, the search yielded 18 studies that met the inclusion criteria. Of these, 15 studies were conducted on COVID-19, 2 studies were on MERS, and 1 study was on SARS. The 15 COVID-19 studies included 538 severe patients (67% male, 33% female) in the final analysis. The one SARS (35) and two MERS studies (36, 37) had fewer patients. The SARS study included 30 severe patients (70% male, 30% female). The two MERS studies (36, 37) had 15 severe patients (80% male, 20% female). The 18 studies also included 1,922 non-severe individuals as controls. Thirteen of the 18 studies were conducted in China, 3 were conducted in South Korea, 1 was conducted in Germany, and 1 was conducted in Ireland. Most of the laboratory tests were conducted after admission.

The characteristics of the included studies are summarized in **Table 1**. Overall, the quality of the included studies was good. The quality assessment score for the included studies ranged from 12 to 18. Most of the studies (94.4%) were of good quality and scored >12 (n = 17), but one study was of moderate quality (12 out of 18). Details regarding the quality assessments are presented in **Table 1** and **Supplementary Table 1**.

**Table 1:** Characteristics of included studies.

Author, year of study (ref)	Diseases	Country	Study type	Severe cases			Non-severe cases			Quality
				Sample size	Age	Study type	Sample size	Age	Male %	
**Chen Guang, 2020** (38)	COVID-19	China	Retrospective study, single center	11	61[IQR 56.5-66]	91% male	10	52.0 [IQR42.8–56.0]	70% male	Good
**Gao Yong, 2020** (39)	COVID-19	China	Retrospective study, single center	15	45.20 ± 7.68	60% males	28	42.96 ± 14.00	60% males	Good
**Han Huan, 2020** (40)	COVID-19	China	Retrospective study, single center	17	65.1 ± 14.4	52% males	42	58.3 ± 12.6	48% males	Moderate
**He Susu, 2020** (41)	COVID-19	China	Retrospective study, single center	33	54 ± 12.5	55% males	60	44 ± 12.5	52% males	Good
**Herold Tobias, 2020** (42)	COVID-19	Germany	Prospective study, single center	13	64 [IQR 45-81]	91% males	27	58 [IQR18-84]	58% males	Good
**Liu Yang, 2020** (43)	COVID-19	China	Retrospective study, single center	30	NA	NA	46	N.A.	NA	Good
**Luo Miao, 2020** (44)	COVID-19	China	Retrospective study, two centers	201	69.00[IQR 62.00–78.00]	66.2% males	817	57.00 [IQR46.0–66.0]	47.5% males	Good
**McElvaney Oliver J, 2020** (45)	COVID-19	Ireland	Retrospective study, single center	20	54.3 ± 18.2	65% males	20	56.6 ± 17.3	60% males	Good
**Chen Ruchong, 2020** (46)	COVID-19	China	Retrospective cohort study, multiple centres	48	61.4 ± 13.6	79% males	354	67.3 ± 12.1	52.8% males	Good
**Wan Suxin, 2020** (47)	COVID-19	China	Cross-sectional, single center	21	61.29 ± 15.55	52% males	102	43.05 ± 13.12	53% males	Good
**Xiaohua Chen, 2020** (48)	COVID-19	China	Retrospective study, single center	17	79.6 ± 12.6	88.2% males	21	52.8 ± 14.2	61.9% males	Good
**Yang A. P, 2020** (49)	COVID-19	China	Retrospective study, single center	24	57.9 ± 11.8	55% males	69	42.1 ± 18.6	75% males	Good
**Yuan X, 2020** (50)	COVID-19	China	Cross-sectional, two centres	46	68 [IQR 61-76]	45.7% males	60	66 [IQR52-69]	49.2% males	Good
**Zhou Yaqing, 2020** (51)	COVID-19	China	Retrospective study, single center	13	67.38 ± 13.36	77% males	8	64.00 ± 15.51	37.5% males	Good
**Zhu Zhe, 2020** (52)	COVID-19	China	Retrospective study, single center	16	57.50 ± 11.70	56.25% males	111	49.95 ± 15.52	65.77% males	Good
**Zhang Yuanchun, 2004** (35)	SARS	China	Retrospective study, single center	30	45.4 [IQR19–86]	70% males	30	44.1 [IQR17–80]	56.6% males	Good
**Hong Ki-Ho, 2018** (36)	MERS	South Korea	Retrospective study, multiple centres	6	59 ± 8	83% males	24	46 ± 13	58% males	Good
**Kim Eu Suk, 2016** (37)	MERS	South Korea	Retrospective study, multiple centres	9	62 ± 13.5	77% males	8	54.25 ± 10.9	75% males	Good

The age is expressed as mean ± S.D. or median [IQR].

### The Level of Circulating Cytokines in COVID-19, MERS, and SARS Patients

The dynamic changes of circulating cytokines (IL-1, IL-2, IL-6, TNF, and IFN-γ, IL-4, IL-10, and IL-17) were analyzed in the 18 included studies (**Table 2**). All the studies reported IL-6 concentrations. Remarkably, all but one study showed a significant elevation in the level of IL-6 in severe COVID-19 patients compared to non-severe groups. The level of circulating IL-6 was also significantly high in severe SARS patients (517 ± 769 pg/ml) compared to non-severe groups (163 ± 796 pg/ml) (35). Furthermore, the two studies (36, 37) that measured the level of circulating cytokines in severe MERS patients showed an elevation in the level of IL-6 in severe MERS patients compared to non-severe groups.

**Table 2:** Details on the proinflammatory and anti-inflammatory cytokines profile among all study participants.

Authors (Ref)	Diseases	Severe	Non-severe	Significant
		ng/ml mean ± SD		
IL-1
**Chen Guang** (38)	COVID-19	5 ± 0.1	5 ± 0.1	No
**McElvaney Oliver J** (45)	COVID-19	40.8 ± 10.4	13.7 ± 5.8	Yes
**Liu Yang** (43)	COVID-19	6.7 ± 2	6.3 ± 1.3	No
**Yang A. P** (49)	COVID-19	38.1 ± 37.4	19.5 ± 12.4	No
IL-2			
**Yuan X** (50)	COVID-19	3.4 ± 0.41	3.5 ± 0.5	No
**Han Huan** (40)	COVID-19	3.4 ± 0.17	3.4 ± 0.23	No
**He Susu** (41)	COVID-19	1.1 ± 0.55	1.4 ± 1.16	No
**Zhu Zhe** (52)	COVID-19	1 ± 0.22	1.04 ± 0.2	No
**Yang A. P** (49)	COVID-19	8.1 ± 6.03	2.25 ± 1.01	Yes
IL-6			
**Yuan X (** 50 **).**	COVID-19	17.3 ± 5.6	9.5 ± 3.1	Yes
**Liu Yang** (43)	COVID-19	37.4 ± 21.8	10.7 ± 5.7	Yes
**Luo Miao** (44)	COVID-19	68.7 ± 18.5	7.2 ± 2.3	Yes
**Chen Guang** (38)	COVID-19	55.5 ± 27	16.6 ± 6.8	Yes
**Xiaohua Chen** (48)	COVID-19	66.4 ± 21.6	13.9 ± 6.8	Yes
**Gao Yong** (39)	COVID-19	38.6 ± 10.5	12.6 ± 4.8	Yes
**Herold Tobias** (42)	COVID-19	158.7 ± 125.5	63.9 ± 52.3	Yes
**Chen Ruchong** (46)	COVID-19	8.7 ± 1.8	7.6 ± 0.7	Yes
**Han Huan** (40)	COVID-19	32.3 ± 16.7	6.7 ± 0.9	Yes
**He Susu** (41)	COVID-19	12.7 ± 8.2	4.6 ± 4.3	Yes
**Zhu Zhe** (52)	COVID-19	26 ± 13.4	4.9 ± 1.3	Yes
**Wan Suxin** (47)	COVID-19	37.8 ± 3.9	13.4 ± 0.6	Yes
**McElvaney Oliver J** (45)	COVID-19	169.4 ± 35.4	45.9 ± 12.4	Yes
**Zhou Yaqing** (51)	COVID-19	17.2 ± 5.6	35.3 ± 1.9	No
**Yang A. P** (49)	COVID-19	326.5 ± 299.4	30.8 ± 27.4	Yes
**Hong Ki-Ho** (36)	MERS	85.3 ± 66.9	12.5 ± 13.8	Yes
**Kim Eu Suk** (37)	MERS	157 ± 38.3	29.5 ± 18.5	Yes
**Zhang Yuanchun** (35)	SARS	517 ± 769	163 ± 796	Yes
IL-17			
**Wan Suxin** (47)	COVID-19	1.16 ± 0.03	1.1 ± 0.01	No
**Yang A. P** (49)	COVID-19	3.4 ± 2.5	3.8 ± 2.5	No
TNF			
**Chen Guang** (38)	COVID-19	10.55 ± 0.4	7.4 ± 0.8	Yes
**Han Huan** (40)	COVID-19	8.7 ± 2.6	5.2 ± 0.4	Yes
**He Susu** (41)	COVID-19	1.13 ± 0.3	1.4 ± 0.5	No
**Luo Miao** (44)	COVID-19	11.3 ± 1.7	6.9 ± 0.5	Yes
**Wan Suxin** (47)	COVID-19	2.9 ± 0.2	4.1 ± 0.5	No
**Yuan X (** 50 **).**	COVID-19	5.1 ± 1.6	4.4 ± 0.8	No
**Zhu Zhe** (52)	COVID-19	1.5 ± 0.1	1.4 ± 0.1	No
**Yang A. P** (49)	COVID-19	284.2 ± 266.3	17.7 ± 13.6	No
**Zhang Yuanchun** (35)	SARS	57.8 ± 5.7	60.1 ± 4.4	No
INF-γ			
**Han Huan** (40)	COVID-19	3.4 ± 0.3	3.3 ± 0.4	No
**He Susu** (41)	COVID-19	2.1 ± 0.6	1.9 ± 0.6	No
**Wan Suxin** (47)	COVID-19	6.9 ± 0.6	5.1 ± 0.3	No
**Yuan X (** 50 **).**	COVID-19	2.9 ± 0.4	2.9 ± 0.4	No
**Zhu Zhe** (52)	COVID-19	1.9 ± 0.3	1.2 ± 0.1	Yes
**Yang A. P** (49)	COVID-19	42.2 ± 37.7	19 ± 16.8	No
**Zhang Yuanchun** (35)	SARS	86.5 ± 20.4	63 ± 20.4	No
IL-4			
**Yuan X (** 50 **).**	COVID-19	2.9 ± 0.5	2.9 ± 0.44	No
**Han Huan** (40)	COVID-19	3.3 ± 0.2	3.4 ± 0.2	Yes
**He Susu** (41)	COVID-19	1.5 ± 0.3	1.7 ± 0.4	No
**Zhu Zhe** (52)	COVID-19	1.2 ± 0.4	1.9 ± 0.19	No
**Wan Suxin** (47)	COVID-19	1.8 ± 0.1	1.7 ± 0.02	No
**Yang A. P** (49)	COVID-19	1.8 ± 0.7	2.7 ± 1.7	No
**Zhang Yuanchun** (35)	SARS	110 ± 12	109 ± 13	No
IL-10			
**Chen Guang** (38)	COVID-19	10.8 ± 0.61	5.8 ± 1.03	Yes
**Han Huan** (40)	COVID-19	8.7 ± 2.6	5.2 ± 0.39	Yes
**He Susu** (41)	COVID-19	4.9 ± 2.6	3.5 ± 0.9	Yes
**Luo Miao** (44)	COVID-19	10 ± 1.7	5.6 ± 0.3	Yes
**McElvaney Oliver J** (45)	COVID-19	47.3 ± 8.7	54.7 ± 7.9	No
**Wan Suxin** (47)	COVID-19	4.6 ± 0.2	2.5 ± 0.03	Yes
**Liu Yang** (37)	COVID-19	7.7 ± 1.6	5.22 ± 0.22	No
**Yuan X (** 50 **).**	COVID-19	4.8 ± 0.6	4.3 ± 0.5	No
**Zhu Zhe** (52)	COVID-19	6.8 ± 1.9	3.3 ± 0.4	Yes
**Yang A. P** (49)	COVID-19	12.6 ± 9.6	3.9 ± 2.5	Yes
**Zhang Yuanchun** (35)	SARS	49.7 ± 12.3	47 ± 5.3	No

Other cytokines were substantially elevated in patients with severe COVID-19, but not in patients with severe SARS and MERS. For example, three studies (38, 40, 44) out of eight showed a significant elevation on TNF level in severe COVID-19 patients compared to non-severe groups. The concentration level of IL-10 was measured in eleven studies (39–41, 47, 49, 52), of which seven studies reported a significant difference between severe COVID-19 patients compared to non-severe groups. In contrast (35), and (36, 37) showed that the level of TNF and IL-10 cytokines were not significantly elevated in patients with severe SARS and MERS, respectively, compared to a non-severe group.

Most of the other cytokines were not comparatively high in severe patients with COVID-19, SARS, and MERS. One study (52) out of six showed that the level of IFN-γ was significantly higher in patients with severe COVID-19 (1.9 ± 0.3 pg/ml) compared to non-severe groups (1.2 ± 0.1 pg/ml). However, another study showed that IFN-γ concentrations were not elevated in patients with severe SARS compared to the non-severe group (35).

Similar results were seen for IL-4. One study (40) out of six showed that the level of IL-4 in severe COVID-19 patients was significantly higher (3.3 ± 0.2 pg/ml) than in non-severe groups (3.4 ± 0.2 pg/ml). However (35), reported that the IL-4 concentration in patients with severe SARS was (110 ± 12 pg/ml), similar to the concentration for a non-severe group (109 ± 13 pg/ml). The levels of IFN-γ and IL-4 were not available in any MERS studies.

The concentrations of IL-1, IL-2, and IL-17 were reported in only four COVID-19 studies (38, 43, 45, 49). Of these, one study (45) showed that the level of IL-1 was significantly higher in patients with severe COVID-19 (40.8 ± 10.4 pg/ml) compared to non-severe groups (13.7 ± 5.8 pg/ml). Similar results were seen for IL-2. One study (49) out of five reported a significant difference between severe COVID-19 patients (8.1 ± 6.03 pg/ml) and non-severe groups (2.25 ± 1.01 pg/ml). The only two studies (47, 49) that measured the level of IL-17 in severe COVID-19 patients showed no significant difference between severe COVID-19 patients and non-severe groups. Altogether, our data suggest that high levels of circulating IL-6, TNF, and IL-10 might be associated with the severity of COVID-19, while only an increased level of IL-6 might be related to the severity of SARS and MERS.

### The Level of Circulating Chemokines in COVID-19, MERS, and SARS Patients

The concentration of the circulating inflammatory chemokine IL-8 were reported in five (35, 38, 43–45, 49) COVID-19 studies and one (35) SARS study. Remarkably, four (38, 43–45, 49) of the five COVID-19 studies reported a significant elevation in the level of IL-8 in severe COVID-19 patients compared to non-severe groups. In contrast, the one study (35) conducted in SARS patients reported a significant reduction in the level of IL-8 in severe SARS patients (143 ± 41 pg/ml) compared to the non-severe group (165 ± 51 pg/ml).

The concentration level of C-X-C motif chemokine 10/interferon-gamma-induced protein 10 (CXCL10/IP10) and chemokine ligand 2/monocyte chemoattractant protein-1 (CCL2/MCP-1) were only reported in two MERS studies (36, 37). The concentrations of CXCL10/IP10 and CCL2/MCP-1 were significantly higher in patients with severe MERS than in non-severe groups.

Although the number of studies measuring the level of chemokines in patients with severe COVID-19, SARS, and/or MERS is limited, the data suggest that circulating IL-8 might be associated with the severity of COVID-19. Moreover, the levels of circulating CXCL10/IP10 and CCL2/MCP-1 might be related to the severity of MERS **(Table 3)**.

**Table 3:** Details on the chemokines profile among all study participants.

Authors (Ref)	Diseases	Severe	Non-severe	Significant
		ng/ml mean ± SD		
IL-8
**Chen Guang** (38)	COVID-19	34.1 ± 9.02	15.8 ± 8.6	no
**Luo Miao** (44)	COVID-19	33.8 ± 6.6	12.9 ± 1.8	Yes
**McElvaney Oliver J** (45)	COVID-19	115.5 ± 23.2	45.2 ± 12	Yes
**Liu Yang** (43)	COVID-19	43.4 ± 30.2	10.2 ± 3.02	Yes
**Yang A. P** (49)	COVID-19	1100.1 ± 994.7	131.63 ± 113.6	Yes
**Zhang Yuanchun** (35)	SARS	143 ± 41	165 ± 51	Yes
CXCL10/IP10				
**Kim Eu Suk** (37)	MERS	2506.8 ± 876.7	327.3 ± 160.4	Yes
**Hong Ki-Ho** (36)	MERS	815.5 ± 230.8	247.8 ± 35.8	Yes
CCL2/MCP-1				
**Hong Ki-Ho** (36)	MERS	888.8 ± 146.8	127.5 ± 26.5	Yes

The 18 studies included in the meta-analysis had a high level of heterogeneity (I^2^ = 99.9%). There was considerable variation between the studies in terms of geographic location, study design, time of blood collection, variable assay used to measure the level of cytokines, and data source for both severe and non-severe cases (**Supplementary Figure 1**).

## Discussion

An emerging body of research is focusing on selective targeting of elevated inflammatory cytokines to treat CRS in hCoV cases (53). However, the mutual cytokine profile of hCoV strains is undetermined. Knowledge of this profile could improve the hyperinflammatory state in critically ill hCoV patients. In addition, there remain unanswered questions about the mechanistic role of the cytokine storm caused by the three hCoVs. Therefore, this systemic review and meta-analysis were performed to analyze the circulatory cytokines and chemokines profiles in patients with severe hCoV infections.

Among the inflammatory parameters, our systematic review and analysis demonstrated a marked elevation in the level of circulating IL-6 and TNF in severe COVID-19 and MERS patients in comparison to non-severe groups. Most of the included studies also showed a significant elevation of circulating IL-10 in severe COVID-19 patients compared to non-severe groups. However, due to the limited number of studies conducted on SARS, we could not find a strong association between high levels of circulating IL-6 and TNF and IL-10 and the severity of disease. This could be the topic of further investigation.

Although clinical data regarding CRS in SARS and MERS infections are limited, an animal study of SARS-CoV has shown that SARS spike protein induced the secretion of high levels of IL-6 and TNF through the NF-kB pathway (54). Others indicated that MERS-infected human monocyte derived-macrophages induce the secretion of high levels of TNF and IL-6 (55). It has also been shown that the coronavirus envelope (E) proteins of SARS-CoV-2 and SARS-CoV share 95% homology (53). It is therefore reasonable to speculate that hCoVs induce similar immune responses.

Among the excessively produced cytokines, IL-6 is considered a key cytokine and an early marker of morbidity and mortality in lung diseases (35, 53, 56)56). For example, a recent study reported the significant role of IL-6 as an independent COVID-19 risk factor using multivariate logistic regression analyses (52). Several large retrospective cohort studies also reported that the level of blood IL-6 correlates with severity and mortality in patients with COVID-19 (57, 58). Evidence suggests that the considerable elevation of IL-6 cytokines in severe COVID-19 patients is linked to massive mucus production by stimulating the expression of the two predominant mucin genes (MUC5AC and MUC5B) in tracheobronchial epithelial cells (59, 60). Recent studies have also revealed that excessive IL-6 signaling leads to hypercoagulating, megakaryocyte activation, precipitation of pulmonary immune-mediated thrombosis (61–63), induction of macrophage activation syndrome, and reduction of myocardium contractility, which might contribute to organ damage (57, 64). Given that IL-6 has a key role in CRS, the recombinant human IL-6 monoclonal antibody tocilizumab is currently the subject of clinical trials, and it shows promising efficacy for the treatment of severe COVID-19 (65–67).

IL-6 has also been found to be significantly correlated with the anti-inflammatory cytokine IL-10, which may reflect self-protection (68, 69). IL-10 is a pleiotropic cytokine primarily responsible for an anti-inflammatory effect (70). It has been shown to inhibit the synthesis of proinflammatory cytokines, such as TNF and IL-6 (71). Unlike in SARS and MERS patients, high levels of IL-10 have been demonstrated in severe and critically ill COVID-19 patients (72). However, it is unclear how IL-10 could be correlated to the severity of COVID-19. Moreover, prolonged production of endogenous IL-10 following the onset of a cytokine storm could be detrimental for the host during COVID-19 infection. For instance, IL-10 has been known to introduce anergy T cells during viral infection (73). Exhausted T cells (PD-1^+^TIM3^+^CD8^+^) have been found in severe cases of patients infected with SARS-CoV-2 and have been reported to be correlated with high serum IL-10 (74). These results suggest that blocking IL-10 or IL-10R might be beneficial for T cell recovery and the prevention of viral persistence. However, several studies have indicated that a lack of IL-10 leads to better bacterial clearance, a higher survival rate, and limited sepsis-induced immunosuppression (75, 76). Therefore, the use of IL-10 antagonists should be carefully studied in the context of severe hCoV infections.

In the past decade, anti-cytokine therapy has made great strides in managing CRS-related diseases, including acute respiratory distress syndrome (ARDS), autoimmunity, and sepsis (53). Therefore, treating severe COVID-19 cases with anti-cytokine treatments has attracted substantial attention as a beneficial addition to antiviral therapy (63, 77). At least 20 registered clinical trials have examined the ability of various IL-6, IL-1, and IFN-1 antagonists to treat COVID-induced CRS (62). We believe that the use of tocilizumab, an anti-IL‐6R antibody (78), is a promising strategy for treating severe hCoV-induced CRS (79), as it showed promising results in clinical trials (80). However, the focus on anti-cytokine therapy might have led to the opportunity to explore other potential therapies that target the host’s immune system, such as stem cell therapy, transfusion of convalescent plasma, targeting of the microRNA network, and immune-supportive therapy (53, 81–84). It is therefore recommended to combine immunotherapy with antiviral treatment. Early diagnosis of CRS in hCoV patients is also recommended to guide therapeutic decisions (53).

Less is known about the chemokine spectrum in human coronaviruses compared to cytokine production. Thus, it is difficult to determine the importance of chemokines in the disease progression for hCoV diseases. However, upon comparing the chemokine profile of the three hCoVs, it can be concluded that CCL2/MCP-1, CXCL8, and CXCL10/IP10 have vital roles in the pathogenesis of infection and serve as prognostic markers for hCoVs’ severity (55, 85–89). Many cell types secrete CXCL8, also known as IL-8 and CCL2/MCP-1, in response to IL-6 and TNF-mediated cytokines (90, 91). CXCL10/IP10 is also secreted by many cells, including monocytes, endothelial cells, and fibroblasts, in response to IFN-γ (92, 93). These chemokines and others play crucial roles in the pathogenesis of diseases characterized by thrombosis and the recruiting of leukocytes to inflamed tissues (94, 95). It is worth noting that CXCL10/IP10 is absent in healthy individuals. However, it is detected in asymptomatic patients and is directly linked to disease severity (96, 97), suggesting that early detection of chemokines can help control new outbreaks (98). Several clinical investigations involving autopsies have also provided evidence that thrombosis is an important consequence of hCoV diseases (99). These findings indicate that the thrombosis-related indicators (CXCL10/IP10, MCP-1) could be directly involved in different stages of hCoV infection (98) and could be used as potential biomarkers to predict the risk of death in hCoV patients (89, 100). Moreover, antagonists targeting chemokines, or their receptors provided a therapeutic benefit against various diseases. For instance, CCR5 and CXCL10/IP-10 antagonists are currently being validated in clinical trials to treat HIV and cardiovascular diseases, respectively (101–103). Thus, neutralization of certain chemokines is a promising potential therapeutic approach to fight hCoVs, especially in patients with thrombotic events (104, 105).

Our demographic data demonstrated that most severe hCoV cases are among older males, in line with previous studies. It has been previously reported that older people with comorbidities are more vulnerable than young people to developing more advanced and severe human coronavirus-related diseases (106). It is believed that dysregulated innate immunity and deficiency in the adaptive immune response of older people, mostly males, could play an essential role in this phenomenon (106). For instance, a previous study demonstrated that peripheral blood mononuclear cells from males stimulated with influenza and herpes-simplex-1 viruses induced the production of higher levels of IL-10 compared to females (107). Other studies showed that males had higher levels of proinflammatory cytokines (e.g., TNF) and chemokines (e.g., CXCL10/IP-10) following lipopolysaccharide stimulation (108). In contrast, females had a higher number of proliferating T-cells and B-cells *in vitro* and antibody response to influenza vaccination than males (109). Patients with comorbidities, such as diabetes, asthma, and hypertension, are at higher risk of mortality because of the excessive production of inflammatory cytokines, including IL-2R, IL-10, and TNF (110–112). These studies and others highlighted that gender and age-specific innate and adaptive immunity and comorbidity might affect hCoV patients’ inflammation reaction, immunotherapy, and immune response to hCoV vaccination.

## Limitations of the Study

Our study has several limitations. The most important is the observational nature of studies and the significant heterogeneity of the study results. However, high statistical heterogeneity is more frequent in meta-analyses of prevalence and descriptive studies (113). This can be explained by differences in the patient populations, underlying comorbidities and coinfections, variant treatments, follow-up, time of blood collection, control donors, and cytokine detection assays. Another significant limitation is the variability in laboratory assays used to measure the level of serum cytokines, as local laboratories have different normal ranges based on local data. This confounding variable may somewhat undermine our results. Thus, our data should be interpreted accounting for this important limitation.

The number of included studies on SARS and MERS was low compared to the number of included studies on COVID-19. However, this can be explained by the fact that COVID-19 infection constitutes a larger threat to the global population compared to SARS and MERS.

Despite these limitations, our meta-analysis remained consistent with observational data analysis, demonstrating the importance of CRS for worsening outcomes for the three hCoV diseases. It is also worth considering that less noticeable cytokine elevations associated with the three hCoV infections might suggest a regulated or insufficient inflammatory response to overwhelming viral infections, such as IFN-γ (81).

## Conclusions and Future Directions

The immune response and immunopathology of the newly emerging hCoV COVID-19 are somewhat similar to those of SARS and MERS. The three zoonotic viral diseases share clinical outcomes characterized by CRS, for which there is still no specific or effective treatment.

Therefore, there is an urgent need to better understand the role of CRS in the disease progression of hCoVs, as this will provide important insights into the type of immune response or pathophysiological events involved in these diseases, which can help to manage the diseases and design new therapies. We recommend using a combination of existing approved therapies with proven safety profiles, such as IL-6 blockade signaling (tocilizumab), for the treatment of hyperinflammation in severe SARS, MERS, and COVID-19 infections. It would also be helpful to measure the inflammatory biomarkers in routine clinical tests to provide an appropriate early risk-based assessment of hCoV patients and thereby reduce the risk of death from hCoV infections. It would also be relevant to determine whether CRS, direct virus-induced tissue damage, or the synergistic effect of both is associated with severe hCoV complications, such as multiple organ dysfunction.

## Data Availability Statement

The original contributions presented in the study are included in the article/**Supplementary Material**. Further inquiries can be directed to the corresponding author.

## Author Contributions

Conception and design: FM. Acquisition of the data: AZ, AN, HA, and FM. Analysis and interpretation of the data: AZ, AN, HA, and FM. Drafting and revising the article: AZ, AN, HA, and FM. All authors contributed to the article and approved the submitted version.

## Conflict of Interest

The authors declare that the research was conducted in the absence of any commercial or financial relationships that could be construed as a potential conflict of interest.
